# Exploited mutualism: the reciprocal effects of plant parasitic nematodes on the mechanisms underpinning plant–mutualist interactions

**DOI:** 10.1111/nph.70125

**Published:** 2025-04-03

**Authors:** Krzysztof Wieczorek, Chris A. Bell

**Affiliations:** ^1^ Department of Agricultural Sciences, Institute of Plant Protection University of Natural Resources and Life Sciences, Vienna 3430 Tulln an der Donau Austria; ^2^ School of Biology, Faculty of Biological Sciences University of Leeds Leeds LS2 9JT UK

**Keywords:** arbuscular mycorrhizal fungi, mutualism, plant parasitic nematodes, plant pathology, symbiosis

## Abstract

We are quickly gaining insights into the mechanisms and functions of plant–mutualist relationships with the common overarching aim of exploiting them to enhance food security and crop resilience. There is a growing mass of research describing various benefits of plant‐mutualistic fungi, including increased nutrition, yields, and tolerance to biotic and abiotic factors. The bulk of this research has been focused on arbuscular mycorrhiza; however, there is now an expansion toward other plant mutualistic fungi. Contrary to the established ‘mycorrhizal induced resistance’ principle, increasing evidence shows that certain plant pests and pathogens may, in fact, exploit the benefits that mutualists provide their hosts, resulting in enhanced pathogenicity and reduced mutualist‐derived benefits. In this Viewpoint, we propose that studying plant mutualistic fungi under controlled artificial conditions indeed provides in‐depth knowledge but may mislead long‐term applications as it does not accurately reflect multi‐symbiont scenarios that occur *in natura*. We summarize the reciprocal impacts of plant pests, such as plant parasitic nematodes, on plant–fungal mutualisms and highlight how glasshouse experiments often yield contradictory results. We emphasize the need for collaborative efforts to increase the granularity of experimental systems, better reflecting natural environments to gain holistic insights into mutualist functions before applying them in sustainable crop protection strategies.

## The role of plant‐mutualistic fungi in food security: from lab to field

The FAO estimates that 20–40% of global crop production is lost due to plant pests and diseases every year (FAO, 2020). The Circular Economy (European Commission, 2020a) and Zero Pollution Action Plan (European Commission, 2021) directives, as well as the Farm to Fork and Biodiversity strategies of the European Union (European Commission, 2020b,c), promote the development of innovative crop protection measures to sustainably enhance and maintain crop yields. Sustainable plant protection strategies are increasingly sought after to reduce fertilizer and chemical pesticide usage in agriculture. Among the promising approaches, the use of mutualistic microorganisms both as biofertilizers and as biological control agents is often studied in varied scenarios.

Mutualistic interactions are those in which two or more species gain reciprocal benefits (Bronstein, 2001). A plant‐based scenario is when mutualistic fungi colonize and exchange nutrients/resources with their plant host, which mutually benefits both organisms. This can increase plant vigor, and for crop plants may increase yields. Crucially, the term ‘plant mutualism’ commonly refers to the direct effects of a single symbiont on a single plant, excluding the indirect effects that the mutualist may have on other plant symbionts, such as pathogens, and knock‐on implications for the host plant or wider plant community. Although this limitation is not inherently problematic, it should be considered when defining the function of a mutualist within a system. While arbuscular mycorrhizal (AM) fungi (van der Heijden *et al*., 2015), and certain *Fusarium* spp. (Ahmed *et al*., 2023), *Trichoderma* spp. (Tseng *et al*., 2020), and *Sebacinales* fungi (Weiß *et al*., 2011) are vastly different in their biology, they all have the potential to form mutualistic relationships with their host plants. This may enhance plant growth, development, and productivity (reviewed in Franken, 2012) while increasing stress tolerance to abiotic factors such as drought, soil acidity, and heavy metals (Porter *et al*., 2020). Arbuscular mycorrhizal fungi are arguably the most well‐studied fungal mutualists of plants, with this classic interaction based on the ‘trade’ of plant carbon to the fungus to support fungal growth, in exchange for AM‐scavenged macro‐ and micronutrients from the soil (Lebreton & Keller, 2024). Although the dynamics of carbon‐for‐nutrient exchange between plants and AM fungi varies between systems and has become somewhat controversial (Bunn *et al*., 2024), it is claimed that their ability to boost the productivity of a vast range of plant species can contribute to future food security and sustainable agriculture (Lebreton & Keller, 2024).

Arbuscular mycorrhizal fungi may increase host resistance and tolerance to various pests and pathogens, such as insect herbivores, fungal and viral pathogens, and plant parasitic nematodes (PPNs) (reviewed in Grabka *et al*., 2022). The mechanisms by which these mutualists antagonize pathogen infection can generally be divided into two modes of action: direct competition for space and nutrition; and indirect effects, such as damage compensation, enhanced tolerance, induced systemic resistance (defense priming), and shifts in root exudation profiles. The priming of plant defense responses by mutualists is an intriguing and well‐researched phenomenon (Cameron *et al*., 2013) consisting of the pre‐activation of systemic plant defense mechanisms before the pathogen's arrival, resulting in an enhanced defense response upon pathogen detection. For instance, priming and subsequent resistance derived from AM fungal colonization (mycorrhizal‐induced resistance (MIR)) may be effective against various pathogens (Kadam *et al*., 2020).

Many of these mechanisms have been studied in the interactions between mutualistic fungi and various PPNs (reviewed in Gianinazzi *et al*., 2010; Vos *et al*., 2012a,b,c, 2013; Daneshkhah *et al*., 2013; Schouteden *et al*., 2015; Poveda *et al*., 2021; Opitz *et al*., 2024). These parasites collectively burden global agriculture by > US$170 billion per annum (Elling, 2013) and are the focus of varied control strategies (Pires *et al*., 2022). Numerous studies indicate their successful and promising use against PPNs. The protective effects of mutualistic fungi often include reduced infection and reproduction rates, as well as enhanced tolerance to nematodes. For example, a seminal study by Vos *et al*. (2012c) demonstrated a mycorrhizal‐induced systemic reduction in root‐knot nematode (*Meloidogyne incognita*) infection. Furthermore, several studies have since highlighted the potential of AM fungi to aid the control of PPNs in evolutionarily diverse crop plants (e.g. Marro *et al*., 2018; Alvarado‐Herrejon *et al*., 2019). Another notable endophyte, *Serendipita indica* (a member of *Sebacinales*), has also been shown to significantly antagonize PPNs and various other plant pathogens (reviewed in Gill *et al*., 2016). For example, during its biotrophic colonization stage, *S. indica* significantly reduces populations of the sugar beet cyst nematode *Heterodera schachtii* (Daneshkhah *et al*., 2013) and *M. incognita* (Opitz *et al*., 2024), leading to disrupted nematode development in *Arabidopsis thaliana*. Although *S. indica* is dissimilar to AM fungi in many regards, its similar effects on co‐occurring PPN populations present an interesting opportunity to determine and investigate conserved phenomena.

Overall, this highlights the vast benefits that plant mutualistic fungi can potentially provide their hosts, not only by directly promoting growth but also by protecting against pathogens such as PPNs. Although most of the above research is laboratory‐ and glasshouse‐based, an optimistic yet potentially incorrect assumption is often globally maintained that the benefits of host–AM interactions translate directly to field soils (Ryan & Graham, 2018). Furthermore, applying Arbuscular Mycorrhizal Fungi inocula without accounting for their persistence, field efficacy, host compatibility, soil conditions, and interactions with resident microbial communities largely overlooks essential ecological principles. Therefore, in this Viewpoint, after years of research articles on the promising role of mutualistic fungi in integrated PPN management strategies and boosting yields of important crop species, we now venture into a critical debate on the use of these mutualists as biocontrol agents. This is fueled by recent research on fungal mutualists affecting PPNs, discussed below, that suggests that these organisms may not always be as beneficial as thought previously. However, while we predominantly focus here on their role in crop defense against pathogens, the broader ecological effects of fungal inocula must also be considered (Vosatka & Dodd, 2002).

## Exploitation of mutualism by plant parasitic nematodes

Although there are clear and significant benefits from plant–AM fungal interactions in certain environments, unfortunately an growing number of risks are increasingly being identified. First, the priming of plant defenses by AM fungi (e.g. MIR) is well documented as being complex, labile, and highly context‐dependent, ultimately impacting host resistance (Schouteden *et al*., 2015; Martinez‐Medina *et al*., 2016; Saikkonen *et al*., 2020). The context‐dependency of MIR has led to ‘mycorrhizal‐induced susceptibility’ (MIS) as an emerging phenomenon (Miozzi *et al*., 2019), whereby mycorrhizal colonization leads to an increase in pathogen populations. Although MIS was initially described in plant‐viral systems, there is now a growing mass of research that has evidenced MIS toward various soil‐borne pests, such as PPNs (Frew *et al*., 2018; Bell *et al*., 2023, 2024; Opitz *et al*., 2024), indicating shared consequences of plant–AM interactions across vastly different pathogens. This raises the question: what determines whether AM–host interactions enhance resistance or susceptibility?

It is logical that a healthier host can support a healthier parasite population, even if this contradicts the idea of mutualistic fungi assisting in plant defense. Several studies have confirmed that AM‐derived nutrients can directly support and enhance the reproductive potential of dramatically different aboveground and belowground pests (Wilkinson *et al*., 2019; Bell *et al*., 2022a,b). The bulk of this research is based on AM–plant interactions; however, MIS is now observed in plant interactions with other mutualistic fungi, such as *S. indica*, despite the dramatic differences between fungal species. Data show the presence of potentially similar underpinning mechanisms, such as enhanced host nutrition and attenuated plant defense responses (Opitz *et al*., 2024). The priming of plant defenses and simultaneous mutualist–host–pathogen nutrient transfer may be independently regulated, underpinning the variable results that are observed between the effects of both mechanisms on pathogen populations.

Although we know that plant‐mutualistic fungi can impact plant pathogens (e.g. MIS), there is still limited knowledge about the reverse effects: what impact do pathogens have on the function of plant mutualists? Studying the function of mutualists, rather than purely their colonization rates, can be challenging but provides direct insights into their role within the host. Phytophagy by aphids or PPNs can dramatically reduce the flow of host resources into the mutualist while the reverse flow of nutrients into the host is maintained (Charters *et al*., 2020; Bell *et al*., 2022a,b, 2024; Durant *et al*., 2023). This highlights an apparent disconnect between both sides of the exchange/interaction (Bunn *et al*., 2024) and the long‐term impact of a reduced resource flow into AM from their pathogen‐infected hosts is unknown. Bell *et al*. (2024) showed that during concurrent phytophagy, cyst nematode‐infected potato maintained fatty acid supply but reduced the flow of hexoses to the AM partner. This may be a direct result of sucrose pool metabolism for plant defense (Wang & Wu, 2023) or simply a matter of symbiont competition. If the pathogen is short‐lived, then the mutualist may be able to survive times of scarce hexose supply by utilizing fatty acids, whereas long‐term biotrophy may be more detrimental for the fungus. Although the relative contributions of plant lipid and hexoses to the fungal carbon economy are unknown (Luginbuehl *et al*., 2017), the inhibition of either has dramatic negative effects (Helber *et al*., 2011; Luginbuehl *et al*., 2017). There are possible similar effects of pathogens on carbon‐for‐nutrient exchange between plants and other, dissimilar mutualists such as *S. indica* (Opitz *et al*., 2021), which are also suggested as new weapons for agricultural security (Saleem *et al*., 2023).

The abundance of pathogens, along with their effects on plant‐fungal carbon flow, may contribute to the dynamic diversity of AM species in the field, both spatially and temporally (van der Heijden *et al*., 2015). Pathogen incidence best predicted the success of AM fungal inoculation in field soils (Lutz *et al*., 2023) and there are links to explore between the mycorrhizal‐community composition and their role in plant defense, beyond resource exchange (Frew *et al*., 2024). These studies show that valuable experimental resolution can be achieved within a ‘broad scope’ experiment to characterize several variables, resulting in field‐relevant data.

Overall, the variable impact of these fungal species indicates the dynamic nature of mutualistic status, which depends on additional factors, such as environmental conditions, plant symbiont genotypes, pathogen identity and virulence, and even co‐evolution of the host, mutualist, and pathogen. Pathogen pressure might also play a role, as it has recently been suggested that a low number of PPNs can, in fact, trigger enhanced plant growth (Topalović & Geisen, 2023), thus being considered mutualistic by the authors, while infection with greater numbers results in the typical detrimental symptoms. This suggests that the distinction between mutualist and pathogen may be thinner than previously thought, emphasizing the limitations of these definitions.

## Promoting field relevance while retaining high‐resolution mutualist–host–pathogen research

The aforementioned plant–mutualist interactions are often explored under the umbrella of agricultural security and may yield beneficial or detrimental outcomes for the plant species of choice, dependent on a range of variables summarized above. Studies using single AM fungal species colonizing a single plant species are then often extrapolated to provide solutions for field‐relevant scenarios (Ryan & Graham, 2018), which may lead to unexpected outcomes, as discussed above. Hence, it will prove valuable to the research community to increase interdisciplinary collaborations between pathology and mutualism researchers to share expertise, test the robustness of growth‐promoting interactions, and better reflect natural systems (Saikkonen *et al*., 2020; Balestrini, 2021; Wippel, 2023; Lebreton & Keller, 2024). Is it worthwhile for researchers from different disciplines to work independently toward the same goal, rather than combining efforts to expedite progress? Despite its benefits, studying multiple, concurrent symbionts does come with a trade‐off, losing the in‐depth resolution gained from single‐symbiont/single‐plant systems. While reduced systems are of great benefit for academic insights, they may not yield suitable applied outcomes and relevance if they omit field scenarios. This is particularly important for projects focused on food security, rather than on academic outputs. Isolated laboratory research may strongly emphasize certain phases in the mutualist's lifecycle while neglecting other aspects such as interactions with the wider soil microbiome and pathobiome. Certainly, it is impossible to explore all potential aboveground and belowground interactions. However, if the goal of research is to improve the vigor of a specific crop, prioritizing the interactions most relevant to the intended environment should be a key focus. The inevitable occurrence of such interactions *in natura* should promote their investigation in academic research, thereby increasing the efficiency of impactful outcomes.

Expediting research toward field‐based experiments or reversing the traditional approach to initiate studies in natural environments may be beneficial for quickly assessing the efficacy of amendments/mutualists in nature, rather than confirming their efficacy in controlled, artificial scenarios. Retrospective studies could then determine the field factors that negated the desired outcomes. Of course, this inevitably would include much more variability and many influencing factors that are not present in controlled glasshouse studies; however, that is precisely why this may be favorable. Studies have shown that this approach can reveal that the co‐presence of pathogens is highly linked to a reduced fungi‐induced benefit (Lutz *et al*., 2023) and also surprisingly highlight that many commercially available fungal inocula that are often used in laboratory studies simply do not colonize in the field through species incompatibility or even nonviable propagules (Salomon *et al*., 2022). This renders their use in glasshouse studies somewhat redundant. Furthermore, if field benefits are the ultimate goal, inoculating soils with multiple, reportedly beneficial, mutualists may be a promising ‘shotgun’ approach leveraging their synergistic effects to enhance plant growth beyond what single inocula can achieve (Afkhami *et al*., 2021). It is known that certain mutualists and pathogens may also enter their host through existing wounds (secondary infection) (Jones *et al*., 2013); therefore, incorporating these possibilities into the experimental system may enhance its field relevance. Furthermore, historically, there has been a separation between researchers studying either the agricultural or ecological relevance of AM fungi. It would seemingly be beneficial to foster collaboration between these different AM fungal disciplines, as the integration of both areas of research would greatly contribute to significant outcomes.

## Conclusions

In summary, the increased susceptibility of fungal‐colonized hosts to pathogens has implications for the role of fungal mutualists in soil amendments, as their actions in natural settings can indirectly lead to significant negative consequences. Similarly, a disruption of plant–AM fungal resource exchange is known to be triggered by pathogens, potentially resulting in long‐term consequences on mutualist populations and function within the wider soil community. A final layer of complexity is added by pathogens potentially providing mutualist‐like benefits at low population densities. Therefore, we summarize that, while numerous studies highlight the positive outcomes of plant–fungal mutualist interactions, these benefits often diminish or even become detrimental when tested in field scenarios or in the presence of field pathogens. This is especially prevalent in the case of PPNs, as recent research demonstrates paradigm shifts, as discussed above. However, it is highly likely that this also applies to other types of plant field pathogens. In this context, it might be incorrect that the prevailing definition of a plant mutualist focuses solely on the one‐on‐one interaction, without necessarily considering the holistic host biome. Therefore, we acknowledge that drawing overall conclusions is challenging, as it requires careful consideration of the interactions and biology of multiple organisms, both among themselves and within highly variable environments. However, to produce robust and field‐relevant research that enhances food security measures, we must collaborate among plant science disciplines and expedite experimental systems‐based research rather than closed artificial environments.

## Competing interests

None declared.

## Author contributions

KW and CAB co‐wrote the manuscript.

## Disclaimer

The New Phytologist Foundation remains neutral with regard to jurisdictional claims in maps and in any institutional affiliations.
